# Factors Associated With Potentially Missed Diagnosis of Appendicitis in the Emergency Department

**DOI:** 10.1001/jamanetworkopen.2020.0612

**Published:** 2020-03-09

**Authors:** Prashant Mahajan, Tanima Basu, Chih-Wen Pai, Hardeep Singh, Nancy Petersen, M. Fernanda Bellolio, Samir K. Gadepalli, Neil S. Kamdar

**Affiliations:** 1 Department of Emergency Medicine, University of Michigan, Ann Arbor; 2 Institute for Healthcare Policy and Innovation, University of Michigan, Ann Arbor; 3 Department of Health Services Research, Baylor College of Medicine, Houston, Texas; 4 Center for Innovations in Quality, Effectiveness and Safety, Michael E. DeBakey Veterans Affairs Medical Center, Houston, Texas; 5 Department of Emergency Medicine, Department of Health Sciences Research, Mayo Clinic, Rochester, Minnesota; 6 Department of Surgery, University of Michigan, Ann Arbor

## Abstract

**Importance:**

Appendicitis may be missed during initial emergency department (ED) presentation.

**Objective:**

To compare patients with a potentially missed diagnosis of appendicitis (ie, patients with symptoms associated with appendicitis, including abdominal pain, constipation, nausea and/or vomiting, fever, and diarrhea diagnosed within 1-30 days after initial ED presentation) with patients diagnosed with appendicitis on the same day of ED presentation to identify factors associated with potentially missed appendicitis.

**Design, Setting, and Participants:**

In this cohort study, a retrospective analysis of commercially insured claims data was conducted from January 1 to December 15, 2019. Patients who presented to the ED with undifferentiated symptoms associated with appendicitis between January 1, 2010, and December 31, 2017, were identified using the Clinformatics Data Mart administrative database (Optum Insights). The study sample comprised eligible adults (aged ≥18 years) and children (aged <18 years) who had previous ED visits within 30 days of an appendicitis diagnosis.

**Main Outcomes and Measures:**

Potentially missed diagnosis of appendicitis. Adjusted odds ratios (AORs) for abdominal pain and its combinations with other symptoms associated with appendicitis were compared between patients with a same-day diagnosis of appendicitis and patients with a potentially missed diagnosis of appendicitis.

**Results:**

Of 187 461 patients with a diagnosis of appendicitis, a total of 123 711 (66%; 101 375 adults [81.9%] and 22 336 children [18.1%]) were eligible for analysis. Among adults, 51 923 (51.2%) were women, with a mean (SD) age of 44.3 (18.2) years; among children, 9631 (43.1%) were girls, with a mean (SD) age of 12.2 (18.2) years. The frequency of potentially missed appendicitis was 6060 of 101 375 adults (6.0%) and 973 of 22 336 children (4.4%). Patients with isolated abdominal pain (adults, AOR, 0.65; 95% CI, 0.62-0.69; *P* < .001; children, AOR, 0.79; 95% CI, 0.69-0.90; *P* < .001) or with abdominal pain and nausea and/or vomiting (adults, AOR, 0.90; 95% CI, 0.84-0.97; *P* = .003; children, AOR, 0.84; 95% CI, 0.71-0.98; *P* = .03) were less likely to have missed appendicitis. Patients with abdominal pain and constipation (adults, AOR, 1.51; 95% CI, 1.31-1.75; *P* < .001; children, AOR, 2.43; 95% CI, 1.86-3.17; *P* < .001) were more likely to have missed appendicitis. Stratified by the presence of undifferentiated symptoms, women (abdominal pain, AOR, 1.68; 95% CI, 1.58-1.78; nausea and/or vomiting, AOR, 1.68; 95% CI, 1.52-1.85; fever, AOR, 1.32; 95% CI, 1.10-1.59; diarrhea, AOR, 1.19; 95% CI, 1.01-1.40; and constipation, AOR, 1.50; 95% CI, 1.24-1.82) and girls (abdominal pain, AOR, 1.64; 95% CI, 1.43-1.88; nausea and/or vomiting, AOR, 1.74; 95% CI, 1.42-2.13; fever, AOR, 1.55; 95% CI, 1.14-2.11; diarrhea, AOR, 1.80; 95% CI, 1.19-2.74; and constipation, AOR, 1.25; 95% CI, 0.88-1.78) as well as patients with a comorbidity index of 2 or greater (adults, abdominal pain, AOR, 3.33; 95% CI, 3.09-3.60; nausea and/or vomiting, AOR, 3.66; 95% CI, 3.23-4.14; fever, AOR, 5.00; 95% CI, 3.79-6.60; diarrhea, AOR, 4.27; 95% CI, 3.39-5.38; and constipation, AOR, 4.17; 95% CI, 3.08-5.65; children, abdominal pain, AOR, 2.42; 95% CI, 1.93-3.05; nausea and/or vomiting, AOR, 2.55; 95% CI, 1.89-3.45; fever, AOR, 4.12; 95% CI, 2.71-6.25; diarrhea, AOR, 2.17; 95% CI, 1.18-3.97; and constipation, AOR, 2.19; 95% CI, 1.30-3.70) were more likely to have missed appendicitis. Adult patients who received computed tomographic scans at the initial ED visit (abdominal pain, AOR, 0.58; 95% CI, 0.52-0.65; nausea and/or vomiting, AOR, 0.63; 95% CI, 0.52-0.75; fever, AOR, 0.41; 95% CI, 0.29-0.58; diarrhea, AOR, 0.83; 95% CI, 0.58-1.20; and constipation, AOR, 0.60; 95% CI, 0.39-0.94) were less likely to have missed appendicitis.

**Conclusions and Relevance:**

Regardless of age, a missed diagnosis of appendicitis was more likely to occur in women, patients with comorbidities, and patients who experienced abdominal pain accompanied by constipation. Population-based estimates of the rates of potentially missed appendicitis reveal opportunities for improvement and identify factors that may mitigate the risk of a missed diagnosis.

## Key Points

**Question:**

What factors are associated with a potentially missed diagnosis of appendicitis in the emergency department among adults and children?

**Findings:**

In this cohort study of 123 711 patients diagnosed with appendicitis, insurance claims data indicated that appendicitis was potentially missed in 6.0% of adults and 4.4% of children during the initial emergency department visit. Factors associated with potentially missed appendicitis included female sex, the coexistence of abdominal pain and constipation, and the presence of comorbidities.

**Meaning:**

Population-based estimates of the rates of potentially missed appendicitis reveal opportunities for improvement and identify factors that may alert clinicians and mitigate the risk of missed diagnosis.

## Teaser

This cohort study uses insurance claims data to compare patients with a potentially missed diagnosis of appendicitis with those diagnosed with appendicitis during the initial emergency department visit to identify factors associated with potentially missed appendicitis among adults and children.

## Introduction

Appendicitis is one of the most common surgical emergencies in the United States. However, the diagnosis of appendicitis is missed in 3.8% to 15.0% of children and in 5.9% to 23.5% of adults during emergency department (ED) visits.^1,2,3,4,5^ Appendicitis is the second most common condition among pediatric patients and the third most common condition cited in adult malpractice insurance claims.^6,7^

The ED is a high-risk setting for diagnostic errors.^8,9^ With approximately 80% of errors potentially preventable^10^ and approximately 50% of all diagnostic errors having the potential for patient harm,^11^ the National Academies of Sciences, Engineering, and Medicine emphasizes that improving the diagnostic process is a public health imperative.^10,11^ A variety of approaches has been used to investigate diagnostic errors,^12^ including a recently proposed conceptual model that uses a symptom-disease dyad approach for analysis.^13^ This approach is particularly useful when there is a biologically plausible association between a symptom or a combination of symptoms and the eventual disease, which, when applied to the clinical context, assumes that the symptom should have directed the practitioner to make a timely and accurate diagnosis.^13^ Symptom-disease dyad analyses have been applied to large data sets to identify potential errors, including claims data errors, and have been used to estimate the probable missed diagnosis of various conditions, including chest pain and myocardial infarction,^14^ dizziness or headache and stroke,^15^ and oncology-related symptoms and pediatric cancer,^16^ to obtain population-level estimates of diagnostic errors.

Previous studies examining patients with appendicitis that was missed at ED visits have tested several factors, including the association of a missed appendicitis diagnosis with an individual symptom or a combination of presenting symptoms, physical examination findings, clinical decision rules, and imaging, specifically abdominal radiography and ultrasonographic imaging in children.^1,2,3^ In other studies, constipation in children, female sex, and patient age (aged <5 years or >50 years) were also associated with a delayed or missed appendicitis diagnosis.^17,18,19^ However, the factors associated with missed appendicitis have been inconsistent, likely owing to small cohort sizes, single-site studies, and loss of patient follow-up, all of which can be mitigated by investigating large administrative data sets. In this study, we analyzed administrative claims data from a large private health insurance provider to examine factors associated with a potentially missed diagnosis of appendicitis at initial ED presentation.

## Methods

We analyzed patients who presented to the ED with undifferentiated symptoms associated with appendicitis between 2010 and 2017 based on a symptom-disease pair analysis of diagnostic error (SPADE) look-back approach.^13^ Patients were identified using Clinformatics Data Mart (Optum Insights), a deidentified administrative claims database that captures all ED, outpatient, and inpatient encounters of more than 75 million individuals who are commercially insured by a single large US private health insurance provider. The database includes member enrollment data, demographic characteristics, individual-level insurance claims, a subset of laboratory test results, hospital discharge information, and pharmacy claims. This study followed the Strengthening the Reporting of Observational Studies in Epidemiology (STROBE) reporting guideline for cohort studies.^20^ The study was approved by the institutional review board of the University of Michigan, with a waiver of consent granted because data were secondary and deidentified.

### Study Population

We identified adults (aged ≥18 years) and children (aged <18 years) who were diagnosed with appendicitis using codes from the *International Classification of Diseases, Ninth Revision, Clinical Modification* (*ICD-9-CM*) and the *International Statistical Classification of Diseases, Tenth Revision, Clinical Modification* (*ICD-10-CM*; eTable 1 in the Supplement).^21,22,23^ We then identified ED visits^24^ among patients who presented with isolated or combinations of undifferentiated symptoms^1,2,3^ that are commonly associated with appendicitis (abdominal pain, constipation, diarrhea, fever, and nausea and/or vomiting) in the 0 to 30 days before an appendicitis diagnosis.^25,26^ If a patient had more than 1 ED visit in a 30-day period with undifferentiated symptoms, we selected the ED visit closest to the appendicitis diagnosis. We excluded patients who had a diagnosis date of appendicitis after the appendectomy date (Figure).

**Figure.**
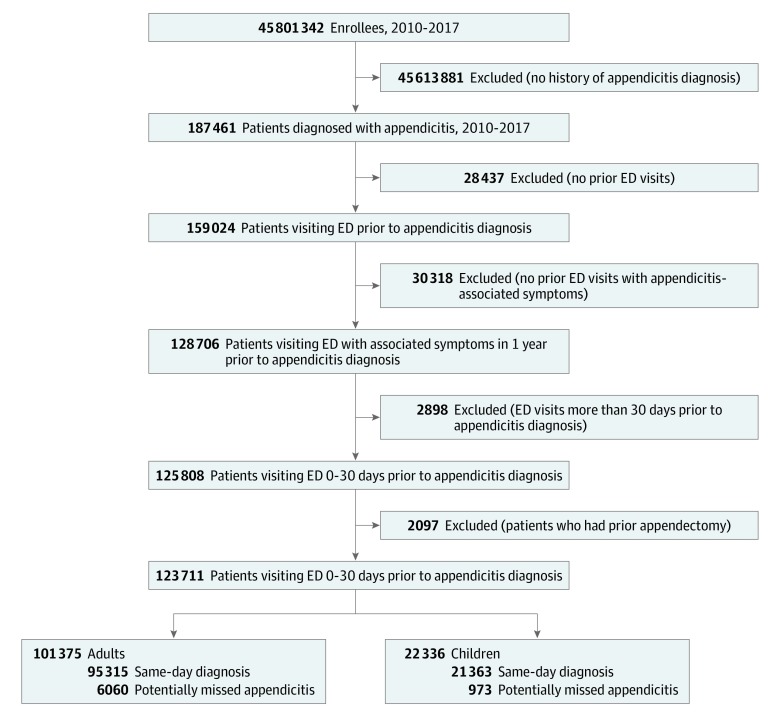
Flow Diagram of Study Population Selection ED indicates emergency department.

We defined a potentially missed diagnosis of appendicitis as an initial (or index) ED visit at which a patient presented with any single undifferentiated symptom or combination of undifferentiated symptoms associated with appendicitis for which the patient did not receive a diagnosis of appendicitis on the same day of symptom presentation but received a subsequent diagnosis of appendicitis within 30 days after the index ED visit (the potentially missed appendicitis group). We treated more than 1 ED visit by the same patient on the same day as a single ED visit. We defined a same-day diagnosis (ie, no missed diagnosis) of appendicitis as an initial ED visit at which a patient presented with any undifferentiated symptom or combination of undifferentiated symptoms associated with appendicitis for which the patient received a diagnosis of appendicitis on the same day of symptom presentation (the same-day diagnosis group).

### Outcome Measure and Covariates

The main outcome of interest was the potentially missed diagnosis of appendicitis. We collected data on covariates from initial ED visits, including patient demographic characteristics (age, sex, race, insurance plan, and US census region)^27^ from health insurance membership files, clinical presentation of symptoms and comorbidities, laboratory tests performed (urinalysis and complete blood cell count), and types of abdominal radiographic imaging performed (radiography, ultrasonography, and computed tomography [CT]; eTable 1 in the Supplement). We calculated the Elixhauser Comorbidity Index to assess the association between potentially missed appendicitis and existing comorbidities for all ED visits.^28^

### Statistical Analysis

We compared the same-day diagnosis and potentially missed appendicitis groups for all categorical variables using χ^2^ tests. Owing to the ordinal nature of the Elixhauser Comorbidity Index, we performed an ordered logistic regression analysis with comorbidity index as the dependent variable and potentially missed appendicitis status as only 1 covariate, and we reported the results of an overall type 3 analysis of fixed effects significance test for the overall effect of potentially missed appendicitis. For continuous variables, such as age, we calculated means and SDs and examined the distribution with the interquartile range (IQR) to ensure robustness of the performance of parametric *t* tests between the 2 groups. Continuous variables were examined and verified for normality using histograms and quantile-quantile plots for visual inspection; therefore, informed by distributional considerations, clinically relevant cutoffs were established. We used Kaplan-Meier survival curves to profile children and adults regardless of the undifferentiated symptom at the index ED visit and the length of time between the index visit and the return visit at which an appendicitis diagnosis was made. We calculated confidence intervals using Hall-Wellner 95% confidence bands and implemented a log-rank test to compare significance between the survival curves of adults and children.

We performed bivariate logistic regression analyses to estimate unadjusted odds ratios (ORs) of potentially missed appendicitis in 6 different models based on the following undifferentiated symptoms as exposure variables: (1) abdominal pain only; (2) abdominal pain and constipation; (3) abdominal pain and nausea and/or vomiting; (4) abdominal pain, nausea and/or vomiting, and fever; (5) abdominal pain, nausea and/or vomiting, fever, and constipation; and (6) no abdominal pain but any of the other undifferentiated symptoms could be present (eMethods in the Supplement). To examine the adjusted odds ratios (AORs) of potentially missed appendicitis for each of the undifferentiated symptom combinations, we developed a family of multivariable logistic regression models with a common set of clinically relevant covariates (demographic characteristics, comorbidities, laboratory tests, and abdominal imaging) and with the aforementioned exposure variable representing a symptom combination covariate.

Diagnostic evaluation for appendicitis was constructed as 1 categorical variable with 4 mutually exclusive groupings: (1) any CT (with or without additional imaging), (2) any ultrasonography (no evidence of CT), (3) radiography only (no evidence of CT or ultrasonography), and (4) no imaging. Further, we used multivariable logistic regression models with the same common set of clinically relevant covariates among 5 subpopulations, each consisting of patients with the presence of 1 single undifferentiated symptom regardless of the presence of other symptoms. Model concordance was examined using a C statistic. We conducted a complete case analysis to examine bias by fitting models with the effective sample size reduction and comparing unadjusted ORs and AORs for each of the included covariates.

Statistical analysis was completed between January 1 and September 15, 2019. All data analyses were conducted using SAS statistical software, version 9.4 (SAS Institute Inc), and statistical testing was 2-tailed and unpaired, with a significance threshold of *P* = .05. All results were reported separately for adults and children.

## Results

Of 187 461 patients with appendicitis diagnosed between January 1, 2010, and December 31, 2017, a total of 123 711 patients (66%; 101 375 adults [81.9%] and 22 336 children [18.1%]) were eligible for analysis. Among adults, 51 923 (51.2%) were women, with a mean (SD) age of 44.3 (18.2) years. Among children, 9631 (43.1%) were girls, with a mean (SD) age of 12.2 (18.2) years. A total of 7033 patients (5.7%) met the criteria for a potentially missed diagnosis of appendicitis, including 6060 adults (6.0%) and 973 children (4.4%) (Figure). The same-day diagnosis group comprised 95 315 adults (94.0%) and 21 363 children (95.6%).

Adults in the potentially missed appendicitis group were older (mean [SD] age, 50.2 [20.0] vs 43.9 [17.9] years, respectively; *P* < .001) and more likely to be women (3884 patients [64.1%] vs 48 039 patients [50.4%], respectively; *P* < .001) (Table 1) compared with adults in the same-day diagnosis group. The 2 adult groups also had different racial compositions, with 506 patients (8.3%) in the potentially missed appendicitis group compared with 5929 patients (6.2%) in the same-day diagnosis group having black ancestry (*P* < .001). Similar differences in sex and race were observed in children. Patients in the potentially missed appendicitis group were also more likely to have 2 or more comorbidities compared with those in the same-day diagnosis group (among adults, 3514 patients [58.0%] vs 29 050 patients [30.5%], respectively; *P* < .001; among children, 121 patients [12.4%] vs 1078 patients [5.0%], respectively; *P* < .001). The appendectomy rate was higher in the same-day diagnosis group compared with the potentially missed appendicitis group among both adults (80 143 patients [84.1%] vs 3097 patients [51.1%], respectively; *P* < .001) and children (17 134 patients [80.2%] vs 667 patients [68.6%], respectively; *P* < .001). Based on the same data shown in Table 1, we calculated the proportion of patients with potentially missed appendicitis for each of the demographic and ED visit characteristics (eTable 2 in the Supplement).

**Table 1.** Demographic and Clinical Characteristics of Patients

Characteristic	Adults, No. (%)			Children, No. (%)		
	Same-Day Diagnosis (n = 95 315)	Potentially Missed Appendicitis (n = 6060)	*P* Value	Same-Day Diagnosis (n = 21 363)	Potentially Missed Appendicitis (n = 973)	*P* Value
Age, mean (SD), y	43.9 (17.9)	50.2 (20.0)	<.001	12.0 (3.7)	11.8 (4.3)	.76
Female sex	48 039 (50.4)	3884 (64.1)	<.001	9098 (42.6)	533 (54.8)	<.001
Race/ethnicity^a^						
White	53 199 (55.8)	3473 (57.3)	<.001	12 281 (57.5)	594 (61.0)	.05
Asian	3071 (3.2)	135 (2.2)		684 (3.2)	29 (3.0)	
Black	5929 (6.2)	506 (8.3)		991 (4.6)	53 (5.4)	
Hispanic	10 767 (11.3)	643 (10.6)		3213 (15.0)	118 (12.1)	
Unknown	22 349 (23.4)	1303 (21.5)		4194 (19.6)	179 (18.4)	
Comorbidity index^b^						
0	46 814 (49.1)	1468 (24.2)	<.001	16 510 (77.3)	598 (61.5)	<.001
1	19 451 (20.4)	1078 (17.8)		3775 (17.7)	254 (26.1)	
≥2	29 050 (30.5)	3514 (58.0)		1078 (5.0)	121 (12.4)	
Imaging type						
CT	88 241 (92.6)	5183 (85.5)	<.001	12 769 (59.8)	630 (64.7)	.002
US	8678 (9.1)	1354 (22.3)	<.001	9296 (43.5)	468 (48.1)	.005
Radiography	10 187 (10.7)	1693 (27.9)	<.001	4017 (18.8)	367 (37.7)	<.001
Laboratory test						
Urinalysis	19 804 (20.8)	1592 (26.3)	<.001	3499 (16.4)	217 (22.3)	<.001
CBC	20 824 (21.8)	2032 (33.5)	<.001	3059 (14.3)	222 (22.8)	<.001
Census region						
Midwest	23 283 (24.4)	1521 (25.1)	.06	5115 (23.9)	269 (27.6)	.03
Northeast	8710 (9.1)	582 (9.6)		1906 (8.9)	70 (7.2)	
South	42 293 (44.4)	2583 (42.6)		9452 (44.2)	435 (44.7)	
West	20 543 (21.6)	1335 (22.0)		4809 (22.5)	195 (20.0)	
Unknown	486 (0.5)	39 (0.6)		81 (0.4)	4 (0.4)	
Commercial insurance	78 486 (82.3)	4066 (67.1)	<.001	21 363 (100)	973 (100)	NA
Appendectomy	80 143 (84.1)	3097 (51.1)	<.001	17 134 (80.2)	667 (68.6)	<.001

Abbreviations: CBC, complete blood cell count; CT, computed tomography; NA, not applicable; US, ultrasonography.

^a^ For race and ethnicity, we used the definitions from the Clinformatics Data Mart database, in which there is only 1 race category, and each appears mutually exclusive. A proprietary algorithm represents a compilation of fields, including known race and proprietary ethnic code tables. A combination of sources, including public records, self-reported surveys, and a proprietary ethnic code table, is used.

^b^ Calculated using the Elixhauser Comorbidity Index.^28^

The frequency of undifferentiated symptoms is presented in eTable 3 in the Supplement. Abdominal pain was the most prevalent symptom in both adults (93 285 patients [97.9%] in the same-day diagnosis group vs 5487 patients [90.5%] in the potentially missed appendicitis group; *P* < .001) and children (20 948 patients [98.1%] the same-day diagnosis group vs 906 patients [93.1%] in the potentially missed appendicitis group; *P* < .001), followed by nausea and/or vomiting. Abdominal pain was more frequently absent in the potentially missed appendicitis group compared with the same-day diagnosis group among both adults (573 patients [9.5%] vs 2020 patients [2.1%], respectively; *P* < .001) and children (67 patients [6.9%] vs 415 patients [1.9%], respectively; *P* < .001). Constipation in combination with abdominal pain was more frequent in the potentially missed appendicitis group compared with the same-day diagnosis group among adults (228 patients [3.8%] vs 1953 patients [2.0%], respectively; *P* < .001) and children (67 patients [6.9%] vs 636 patients [3.0%], respectively; *P* < .001).

Computed tomography was the most common diagnostic radiologic test performed among patients diagnosed with appendicitis regardless of the patient’s age and the timing of the diagnosis (same-day vs return visit); adults were more likely to receive a CT scan than children, with 93 424 adults (92.2%; 88 241 patients [92.6%] in the same-day diagnosis group vs 5183 patients [85.5%] in the potentially missed appendicitis group; *P* < .001) and 13 399 children (60.0%; 12 769 patients [59.8%] in the same-day diagnosis group vs 630 patients [64.7%] in the potentially missed appendicitis group; *P* < .001) receiving a CT scan (Table 1).

Ultrasonography was the second most common imaging modality, performed in 10 032 adults (9.9%; 8678 patients [9.1%] in the same-day diagnosis group vs 1354 patients [22.3%] in the potentially missed appendicitis group; *P* < .001) and 9764 children (43.7%; 9296 patients [43.5%] in the same-day diagnosis group vs 468 patients [48.1%] in the potentially missed appendicitis group; *P* = .005). Unlike the same-day diagnosis group, in which a CT scan was performed at the index ED visit, only 105 of 5183 adults (2.0%) and 13 of 630 children (2.1%) in the potentially missed appendicitis group received a CT scan at the index visit. Indeed, most of the radiologic tests, including CT, ultrasonography, radiography, and laboratory investigations (including complete blood cell count and urinalysis), were performed in the potentially missed appendicitis group at return ED visits and not at the index ED visit (eTable 6 in the Supplement).

Kaplan-Meier survival curves stratified by children and adults show the probability of a patient receiving an appendicitis diagnosis at a return visit after the initial ED visit (eFigure in the Supplement). Children had a significantly shorter median time in the number of days between the index visit and return visit (2 days; IQR, 1-7 days) compared with adults (4 days; IQR, 1-13 days) (log rank, *P* < .001).

The results from logistic regression models are reported in Table 2, which shows unadjusted ORs and AORs for exposure to abdominal pain alone and in combination with other symptoms. Patients who did not have abdominal pain at the index ED presentation were more likely to have missed appendicitis, both in adults (AOR, 3.57; 95% CI, 3.22-3.95; *P* < .001) and children (AOR, 2.99; 95% CI, 2.25-3.96; *P* < .001). Patients who had both abdominal pain and constipation at the index ED visit (in adults, AOR, 1.51; 95% CI, 1.31-1.75; in children, AOR, 2.43; 95% CI, 1.86-3.17) were also more likely to have missed appendicitis. In the 6 adult models, the AORs for black patients compared with white patients consistently ranged from 1.14 to 1.16 (abdominal pain, AOR, 1.14; 95% CI, 1.02-1.27; nausea and/or vomiting, AOR, 1.09; 95% CI, 0.92-1.29; fever, AOR, 1.17; 95% CI, 0.84-1.65; diarrhea, AOR, 0.80; 95% CI, 0.58-1.11; and constipation, AOR, 0.78; 95% CI, 0.55-1.09) (Table 3). This finding was not observed in the 6 pediatric models. The detailed results for all covariates included in the models are reported in eTable 4 and eTable 5 in the Supplement.

**Table 2.** Abdominal Pain and Its Combinations With Other Symptoms of Potentially Missed Appendicitis^a^

Symptom	Unadjusted		Adjusted^**b**^	
	OR (95% CI)	*P* Value	OR (95% CI)	*P* Value
**Adults**				
Total, No.	101 375		100 833	
Abdominal pain only	0.59 (0.56-0.62)	<.001	0.65 (0.62-0.69)	<.001
Abdominal pain and constipation	1.87 (1.63-2.15)	<.001	1.51 (1.31-1.75)	<.001
Abdominal pain and nausea and/or vomiting	0.86 (0.81-0.92)	<.001	0.90 (0.84-0.97)	.003
Abdominal pain, nausea and/or vomiting, and fever	0.77 (0.63-0.94)	.009	0.78 (0.64-0.95)	.02
Abdominal pain, nausea and/or vomiting, fever, and constipation	1.21 (0.61-2.39)	.58	0.94 (0.47-1.87)	.86
No abdominal pain	4.80 (4.36-5.29)	<.001	3.57 (3.22-3.95)	<.001
**Children**				
Total, No.	22 336		22 250	
Abdominal pain only	0.71 (0.62-0.81)	<.001	0.79 (0.69-0.90)	<.001
Abdominal pain and constipation	2.41 (1.86-3.13)	<.001	2.43 (1.86-3.17)	<.001
Abdominal pain and nausea and/or vomiting	0.81 (0.69-0.95)	.01	0.84 (0.71-0.98)	.03
Abdominal pain, nausea and/or vomiting, and fever	0.83 (0.63-1.10)	.20	0.77 (0.58-1.02)	.06
Abdominal pain, nausea and/or vomiting, fever, and constipation	1.45 (0.76-2.76)	.26	1.09 (0.56-2.10)	.80
No abdominal pain	3.73 (2.86-4.87)	<.001	2.99 (2.25-3.96)	<.001

Abbreviation: OR, odds ratio.

^a^ Independent unadjusted and adjusted ORs were calculated, in which the reference group included all patients who were not exposed. For instance, patient episodes in which the patient presented with abdominal pain only would have had a reference group of all others who were not in this undifferentiated symptom combination category.

^b^ Models were adjusted for age group, sex, race/ethnicity, census region, insurance product, comorbidity index, laboratory tests, imaging diagnostic workups, and respective symptom or symptom combination.

**Table 3.** Multivariable Modeling of Potentially Missed Appendicitis Among Adults, Stratified by Presentation of Single Symptom^a^

Variable	AOR (95% CI)				
	Abdominal Pain (n = 98 253)	Nausea/Vomiting (n = 31 787)	Fever (n = 6785)	Diarrhea (n = 7005)	Constipation (n = 4536)
Age group, y					
18-25	1 [Reference]	1 [Reference]	1 [Reference]	1 [Reference]	1 [Reference]
26-40	1.00 (0.91-1.09)	0.93 (0.80-1.07)	0.85 (0.60-1.21)	0.77 (0.59-0.99)	0.88 (0.61-1.27)
41-64	0.82 (0.75-0.90)	0.79 (0.68-0.91)	0.84 (0.60-1.16)	0.52 (0.40-0.69)	0.65 (0.45-0.92)
≥65	0.81 (0.70-0.92)	0.83 (0.67-1.03)	0.86 (0.55-1.34)	0.58 (0.40-0.84)	0.82 (0.53-1.27)
Sex					
Male	1 [Reference]	1 [Reference]	1 [Reference]	1 [Reference]	1 [Reference]
Female	1.68 (1.58-1.78)	1.68 (1.52-1.85)	1.32 (1.10-1.59)	1.19 (1.01-1.40)	1.50 (1.24-1.82)
Race/ethnicity^b^					
White	1 [Reference]	1 [Reference]	1 [Reference]	1 [Reference]	1 [Reference]
Asian	0.75 (0.62-0.90)	0.77 (0.56-1.06)	0.58 (0.31-1.10)	1.21 (0.29-1.99)	0.91 (0.51-1.64)
Black	1.14 (1.02-1.27)	1.09 (0.92-1.29)	1.17 (0.84-1.65)	0.80 (0.58-1.11)	0.78 (0.55-1.09)
Hispanic	0.96 (0.87-1.06)	0.91 (0.77-1.06)	1.00 (0.74-1.35)	1.02 (0.78-1.34)	0.95 (0.69-1.31)
Unknown/missing	0.94 (0.88-1.01)	0.92 (0.83-1.04)	0.86 (0.68-1.08)	0.94 (0.77-1.15)	0.87 (0.69-1.11)
Comorbidity index^c^					
0	1 [Reference]	1 [Reference]	1 [Reference]	1 [Reference]	1 [Reference]
1	1.71(1.57-1.86)	1.83 (1.59-2.10)	1.46 (1.03-2.06)	1.75 (1.34-2.30)	1.84 (1.30-2.61)
≥2	3.33 (3.09-3.60)	3.66 (3.23-4.14)	5.00 (3.79-6.60)	4.27 (3.39-5.38)	4.17 (3.08-5.65)
Imaging type					
No imaging	1 [Reference]	1 [Reference]	1 [Reference]	1 [Reference]	1 [Reference]
Any CT	0.58 (0.52-0.65)	0.63 (0.52-0.75)	0.41 (0.29-0.58)	0.83 (0.58-1.20)	0.60 (0.39-0.94)
Any US, no CT	2.15 (1.81-2.56)	2.33 (1.77-3.09)	1.08 (0.56-2.09)	1.76 (0.93-3.32)	1.67 (0.75-3.73)
Radiography only	2.73 (2.12-3.51)	2.83 (1.93-4.16)	1.49 (0.63-3.52)	3.65 (1.84-7.23)	4.07 (2.11-7.85)
Health insurance					
Medicare	1 [Reference]	1 [Reference]	1 [Reference]	1 [Reference]	1 [Reference]
Commercial	0.78 (0.70-0.87)	0.74 (0.63-0.89)	0.93 (0.67-1.30)	0.71 (0.53-0.95)	0.87 (0.64-1.20)
Census region					
West	[Reference]	[Reference]	[Reference]	[Reference]	[Reference]
Midwest	1.11 (1.02-1.20)	1.10 (0.96-1.26)	0.72 (0.54-0.95)	1.41 (1.11-1.79)	1.33 (1.00-1.77)
Northeast	0.97 (0.80-1.08)	0.94 (0.79-1.13)	0.96 (0.70-1.31)	1.17 (0.87-1.57)	0.88 (0.61-1.27)
South	0.97 (0.90-1.04)	0.91 (0.81-1.03)	0.73 (0.58-0.93)	1.13 (0.91-1.40)	1.10 (0.85-1.42)
Laboratory test					
No test	1 [Reference]	1 [Reference]	1 [Reference]	1 [Reference]	1 [Reference]
Urinalysis	0.95 (0.88-1.03)	1.00 (0.88-1.12)	0.87 (0.70-1.09)	0.96 (0.78-1.18)	0.83 (0.66-1.06)
CBC	1.36 (1.27-1.47)	1.37 (1.22-1.54)	1.49 (1.20-1.84)	1.21 (0.99-1.48)	1.15 (0.91-1.46)
C statistic	0.695	0.713	0.726	0.698	0.695

Abbreviations: AOR, adjusted odds ratio; CBC, complete blood cell count; CT, computed tomography; US, ultrasonography.

^a^ Independent AORs were calculated, in which the reference group included all patients who were not exposed. For instance, patient episodes in which the patient presented with abdominal pain only would have had a reference group of all others who were not in this undifferentiated symptom combination category.

^b^ For race and ethnicity, we used the definitions from the Clinformatics Data Mart database, in which there is only 1 race category, and each appears mutually exclusive. A proprietary algorithm represents a compilation of fields, including known race and proprietary ethnic code tables. A combination of sources, including public records, self-reported surveys, and a proprietary ethnic code table, is used.

^c^ Calculated using the Elixhauser Comorbidity Index.^28^

After stratifying for symptom presentation, women (abdominal pain, AOR, 1.68; 95% CI, 1.58-1.78; nausea and/or vomiting, AOR, 1.68; 95% CI, 1.52-1.85; fever, AOR, 1.32; 95% CI, 1.10-1.59; diarrhea, AOR, 1.19; 95% CI, 1.01-1.40; and constipation, AOR, 1.50; 95% CI, 1.24-1.82) and adults with 2 or more comorbidities (abdominal pain, AOR, 3.33; 95% CI, 3.09-3.60; nausea and/or vomiting, AOR, 3.66; 95% CI, 3.23-4.14; fever, AOR, 5.00; 95% CI, 3.79-6.60; diarrhea, AOR, 4.27; 95% CI, 3.39-5.38; and constipation, AOR, 4.17; 95% CI, 3.08-5.65) were more likely to have missed appendicitis (Table 3). Furthermore, adults who received a CT scan (abdominal pain, AOR, 0.58; 95% CI, 0.52-0.65; nausea and/or vomiting, AOR, 0.63; 95% CI, 0.52-0.75; fever, AOR, 0.41; 95% CI, 0.29-0.58; diarrhea, AOR, 0.83; 95% CI, 0.58-1.20; and constipation, AOR, 0.60; 95% CI, 0.39-0.94) were less likely to have missed appendicitis. However, adults who underwent radiography as the only radiologic test (abdominal pain, AOR, 2.73; 95% CI, 2.12-3.51; nausea and/or vomiting, AOR, 2.83; 95% CI, 1.93-4.16; fever, AOR, 1.49; 95% CI, 0.63-3.52; diarrhea, AOR, 3.65; 95% CI, 1.84-7.23; and constipation, AOR, 4.07; 95% CI, 2.11-7.85) were more likely to have missed appendicitis.

Similar results were observed among children, with girls (abdominal pain, AOR, 1.64; 95% CI, 1.43-1.88; nausea and/or vomiting, AOR, 1.74; 95% CI, 1.42-2.13; fever, AOR, 1.55; 95% CI, 1.14-2.11; diarrhea, AOR, 1.80; 95% CI, 1.19-2.74; and constipation, AOR, 1.25; 95% CI, 0.88-1.78) and children with 2 or more comorbidities (abdominal pain, AOR, 2.42; 95% CI, 1.93-3.05; nausea and/or vomiting, AOR, 2.55; 95% CI, 1.89-3.45; fever, AOR, 4.12; 95% CI, 2.71-6.25; diarrhea, AOR, 2.17; 95% CI, 1.18-3.97; and constipation, AOR, 2.19; 95% CI, 1.30-3.70) more likely to have missed appendicitis (Table 4).

**Table 4.** Multivariable Modeling of Potentially Missed Appendicitis Among Children, Stratified by Presentation of Single Symptom^a^

Variable	AOR (95% CI)				
	Abdominal Pain (n = 21 770)	Nausea/Vomiting (n = 8994)	Fever (n = 3790)	Diarrhea (n = 1696)	Constipation (n = 1679)
Age group, y					
0-5	1 [Reference]	1 [Reference]	1 [Reference]	1 [Reference]	1 [Reference]
6-10	0.59 (0.45-0.78)	0.49 (0.34-0.70)	0.51 (0.34-0.78)	0.34 (0.17-0.66)	1.16 (0.60-2.27)
11-15	0.62 (0.48-0.80)	0.46 (0.32-0.64)	0.48 (0.32-0.73)	0.32 (0.17-0.60)	1.32 (0.69-2.53)
16-17	0.68 (0.51-0.89)	0.57 (0.39-0.83)	0.70 (0.41-1.19)	0.32 (0.16-0.66)	1.38 (0.66-2.89)
Sex					
Male	1 [Reference]	1 [Reference]	1 [Reference]	1 [Reference]	1 [Reference]
Female	1.64 (1.43-1.88)	1.74 (1.42-2.13)	1.55 (1.14-2.11)	1.80 (1.19-2.74)	1.25 (0.88-1.78)
Race/ethnicity^b^					
White	1 [Reference]	1 [Reference]	1 [Reference]	1 [Reference]	1 [Reference]
Asian	0.89 (0.59-1.33)	1.02 (0.57-1.84)	1.31 (0.63-2.71)	0.97 (0.28-3.35)	1.13 (0.47-2.75)
Black	1.04 (0.76-1.41)	0.99 (0.62-1.58)	0.79 (0.37-1.68)	0.52 (0.15-1.72)	0.91 (0.42-1.97)
Hispanic	0.73 (0.59-0.91)	0.91 (0.67-1.23)	0.82 (0.52-1.30)	1.05 (0.60-1.85)	0.68 (0.38-1.20)
Unknown/missing	0.89 (0.74-1.06)	1.03 (0.79-1.33)	0.99 (0.65-1.49)	1.01 (0.58-1.77)	0.99 (0.63-1.55)
Comorbidity index^c^					
0	1 [Reference]	1 [Reference]	1 [Reference]	1 [Reference]	1 [Reference]
1	1.76 (1.50-2.06)	1.86 (1.48-2.34)	2.36 (1.66-3.34)	2.34 (1.48-3.68)	2.08 (1.38-3.13)
≥2	2.42 (1.93-3.05)	2.55 (1.89-3.45)	4.12 (2.71-6.25)	2.17 (1.18-3.97)	2.19 (1.30-3.70)
Imaging type					
No imaging	1 [Reference]	1 [Reference]	1 [Reference]	1 [Reference]	1 [Reference]
Any CT	1.08 (0.85-1.36)	0.97 (0.69-1.37)	0.83 (0.52-1.35)	0.58 (0.30-1.10)	1.34 (0.47-3.82)
Any US, no CT	0.78 (0.60-1.01)	0.69 (0.47-1.02)	0.65 (0.38-1.13)	0.36 (0.17-0.79)	1.01 (0.34-3.01)
Radiography only	1.69 (1.09-2.61)	1.84 (0.98-3.44)	1.36 (0.52-3.55)	0.37 (0.08-1.84)	1.17 (0.34-4.02)
Census region					
West	1 [Reference]	1 [Reference]	1 [Reference]	1 [Reference]	1 [Reference]
Midwest	1.16 (0.95-1.42)	1.32 (0.97-1.80)	0.98 (0.62-1.55)	1.41 (0.74-2.68)	1.16 (0.65-2.07)
Northeast	0.92 (0.69-1.22)	1.27 (0.83-1.93)	0.69 (0.36-1.35)	1.38 (0.61-3.11)	0.56 (0.22-1.40)
South	1.07 (0.89-1.28)	1.16 (0.88-1.52)	0.91 (0.62-1.35)	1.13 (0.64-1.98)	1.30 (0.79-2.13)
Laboratory test					
No test	1 [Reference]	1 [Reference]	1 [Reference]	1 [Reference]	1 [Reference]
Urinalysis	1.06 (0.88-1.29)	1.19 (0.91-1.57)	1.24 (0.84-1.83)	1.30 (0.76-2.24)	0.96 (0.58-1.59)
CBC	1.56 (1.29-1.88)	1.83 (1.40-2.39)	1.42 (0.96-2.09)	2.10 (1.24-3.56)	1.12 (0.67-1.89)
C statistic	0.642	0.674	0.699	0.711	0.636

Abbreviations: AOR, adjusted odds ratio; CBC, complete blood cell count; CT, computed tomography; US, ultrasonography.

^a^ Independent AORs were calculated, in which the reference group included all patients who were not exposed. For instance, patient episodes in which the patient presented with abdominal pain only would have had a reference group of all others who were not in this undifferentiated symptom combination category.

^b^ For race and ethnicity, we used the definitions from the Clinformatics Data Mart database, in which there is only 1 race category, and each appears mutually exclusive. A proprietary algorithm represents a compilation of fields, including known race and proprietary ethnic code tables. A combination of sources, including public records, self-reported surveys, and a proprietary ethnic code table, is used.

^c^ Calculated using the Elixhauser Comorbidity Index.^28^

## Discussion

We analyzed 8 years of insurance claims from a large private health insurance provider to estimate the frequency of a potentially missed diagnosis of appendicitis and to describe the associated factors. We found that female sex and a higher comorbidity index were associated with potentially missed appendicitis in both children and adults. Patients with a potentially missed diagnosis of appendicitis were more likely to be examined using only abdominal radiography during the initial ED visit. Among adults, black race was associated with potentially missed appendicitis in models that included isolated symptoms or symptom combinations, but this association was not seen in children.

The analysis of administrative data sets allowed us to draw estimates of missed appendicitis diagnoses in large cohorts of patients. Abdominal pain, the most common presenting symptom with or without associated symptoms (constipation, diarrhea, fever, and nausea and/or vomiting), is closely associated with appendicitis. However, abdominal pain is also a major factor associated with return ED visits. One ED-based study examining return visits among patients with abdominal pain revealed that nearly one-third of cases had a diagnostic error.^29^ Our approach of using large administrative data sets enabled us to overcome sample size limitations and could account for the loss of patients seeking care outside the original health care system after the initial ED visit; thus, this approach offered additional insights into the epidemiology of diagnostic errors.^30,31^

Our study findings of missed appendicitis rates for adults (6.0%) and children (4.4%) along with the increased risk in women, older adults, and individuals with comorbid conditions are consistent with previous studies, thus suggesting the validity of the approach used in this study.^32,33^ In addition, only 1 previous study revealed appendicitis as the most common missed diagnosis among children with constipation,^17^ which is consistent with our results. Constipation at index ED presentation may be a factor in the decision-making of the ED practitioner and may add to the risk of false-negative test results for appendicitis. However, no such data exist in adults with a missed diagnosis of appendicitis. Constipation might be the reason that practitioners perform radiography, which may lead to confirmation bias and early closure in the medical decision-making process, even though the utility of radiography for constipation is unclear.^34,35^

The increased risk of potentially missed appendicitis associated with race is a finding requiring further exploration. In 6 separate multivariable models involving abdominal pain or its combination with other symptoms along with other variables, black race compared with white race was consistently associated with a higher rate of potentially missed appendicitis among adults, while adult Asian and pediatric Hispanic patients were less likely to have missed appendicitis (eTable 4 and eTable 5 in the Supplement). Although observations of racial and ethnic disparities in health care in general and in ED care in specific are not new,^36,37^ only 1 previous study of missed appendicitis included race in the analysis, and it did not find race to be associated with missed appendicitis.^2^ Our results identified racial disparity in a group of privately insured patients in a single-payer claims data set. Future work should involve validating the disparities in care associated with race and/or ethnicity in additional data sets, including Medicaid and Medicare claims data, and other types of prospectively conducted studies designed to investigate these differences.

Our analyses of radiologic investigations performed at the initial ED visit revealed discordance between guidelines and appropriate use. Ultrasonographic imaging is inexpensive, does not expose the patient to ionizing radiation, and is the recommended first-line imaging modality in children and pregnant women.^38,39^ However, ultrasonographic imaging is operator dependent, and the appendix is often difficult to visualize in pregnant women, obese patients, and patients with retrocecal appendices.^38,40^ Computed tomography exposes patients to ionizing radiation and is often considered a second-line imaging modality, especially in children. In our analyses, adult patients with undifferentiated symptoms were more likely to be diagnosed with appendicitis at initial presentation if they had CT performed. For adults, the ultrasonography rate of 22.3% in the potentially missed appendicitis group and 9.1% in the same-day diagnosis group may reflect the lower test performance of ultrasonography compared with CT as an imaging modality. In children, the overall lower CT rates in both the same-day diagnosis and potentially missed appendicitis groups and the higher ultrasonography rates are consistent with the emphasis on less exposure to ionizing radiation in children.^38,39^ Patients who received only plain abdominal radiography were more likely to be in the potentially missed appendicitis group regardless of age. This finding is consistent with the suboptimal test characteristics of plain radiography in the diagnosis of appendicitis^41^ and likely reflects the possibility that the clinicians were not considering appendicitis as the primary diagnosis.

With additional validation, our results may be able to assist clinicians by identifying phenotypes in patients for whom missed appendicitis is likely. Notably, our analysis could help further improve clinical decision-making by defining populations at risk of potentially missed appendicitis, and it could lead to a more careful discussion with the family and patient with regard to the further need for imaging, the type and timing of imaging, and follow-up. For instance, if validated, our results suggest that the probability of missed appendicitis for an Asian man aged 41 to 60 years who presents to the ED with abdominal pain and no comorbid conditions is 2.8%. On the other hand, a black woman aged 18 to 25 years with abdominal pain and 2 or more comorbid conditions would have a probability of missed appendicitis of 22.9%. Such estimates, if validated, are actionable and can be incorporated during clinical evaluation to enhance shared decision-making and ensure access to follow-up health care services as well as safe and timely diagnosis.^10^

### Limitations

Our analysis had several limitations. Administrative claims data are limited by variability in the assignment of diagnosis codes. For instance, the appendectomy rates in our analysis were lower than the reported rates (>90%) of other studies,^41,42,43,44^ especially among the potentially missed appendicitis group, which may be reflective of incomplete coding or billing. A low appendectomy rate may also reflect a mislabeling in the claims data of patients with an appendicitis diagnosis. In this study, we took a more liberal approach to defining appendicitis with the aim of overcoming possible coding errors in claims data. Future studies may develop an optimal definition for the diagnosis of appendicitis in administrative data. The use of claims data without robust patient-level clinical data and context did not allow us to draw conclusions about the frontline decision-making or system factors involved in the diagnostic process.^45^ However, this approach allowed us to identify signals in large data sets to screen for patients at a higher risk of potentially missed appendicitis.

We used a simplistic symptom-disease dyad approach and added a multisymptom-disease nuance to draw our conclusions. Although we used the look-back approach, we cannot comment on whether our findings would be different if both approaches (ie, look-back and look-forward) were simultaneously applied. Although similar symptom-disease dyad methods have been used in analyses of administrative data sets to gauge estimates about diagnostic errors,^13,14,15^ future studies using chart abstraction on patient-level data, such as the presence of right lower-quadrant pain or rebound tenderness as a criterion standard,^46^ are still needed to validate the claims-based definition for missed appendicitis. To better understand the frequency of potentially missed appendicitis among patients who present with uncommon and atypical symptoms as well as the role of practitioner characteristics, we suggest that our results should be validated using a study design that incorporates medical record review. Laboratory tests, especially complete blood cell counts, are often used in the diagnostic process for appendicitis, but very few laboratory claims (21%) were available, which was likely owing to incomplete laboratory claims data, bundled payments, and missing incorporation of laboratory coding.

Among patients who received a CT scan at the index ED visit, 5.5% of adults and 4.7% of children were in the potentially missed appendicitis group. It is difficult to determine whether the radiologist or ED physician missed the signs of appendicitis at the index ED visit or whether the patient had normal CT results at the index ED visit followed by an appendicitis diagnosis at a return visit. Although a CT scan of the abdomen of patients with appendicitis has been reported to have excellent performance characteristics,^47,48^ 2% of patients (105 adults and 13 children) with potentially missed appendicitis in our study had received a CT scan at the index visit. We are unable to comment on whether these results represent patients with unrelated ED visits (ie, the CT indicated true-negative results and the patient did not have appendicitis vs the patient had potentially missed appendicitis owing to false-negative CT results). All of these limitations can be overcome by future studies incorporating patient-level record reviews.^49^ Our data were limited to 1 private insurance provider; therefore, the results may not be representative of the entire US population. However, the advantage of using a single-payer claims data set included a lower likelihood of missing data, especially among patients who may not have had a return visit to the same ED.

## Conclusions

Regardless of age, a missed diagnosis of appendicitis was more likely to occur in female patients, patients with comorbidities, and patients with abdominal pain accompanied by constipation. Population-based estimates of the rates of potentially missed appendicitis reveal opportunities for improvement and identify factors that may alert clinicians and mitigate the risk of missed diagnosis.

## Notes

Supplement. **eMethods.** SAS Codes **eFigure.** Kaplan-Meier Curves for Days From Index Emergency Department Visit to Appendicitis Diagnosis Stratified by Adults and Children With 95% Hall-Wellner Bands **eTable 1.** Diagnosis and Procedure Codes **eTable 2.** Percent of Potentially Missed Appendicitis for Demographic and Visit Characteristics **eTable 3.** Frequency of Clinical Symptoms and Combinations of Symptoms in Patients With Same Day Diagnosis Compared With Those with Potentially Missed Appendicitis **eTable 4.** Adults—Multivariable Modeling of Potentially Missed Appendicitis With Abdominal Pain and Its Combination With Other Symptom(s) Included Along with Other Covariates (N = 100 833) **eTable 5.** Children—Multivariable Modeling of Potentially Missed Appendicitis With Abdominal Pain and Its Combination with Other Symptom(s) Included Along With Other Covariates (N = 22 250) **eTable 6.** Potentially Missed Appendicitis Cases That Had Imaging at Index Emergency Department Visit Compared With Those That Had Imaging on Return Visit
