# Supplementary material for: Factors Associated With Potentially Missed Diagnosis of Appendicitis in the Emergency Department
Source: JAMA Netw Open. 2020 Mar 9;3(3):e200612. doi: 10.1001/jamanetworkopen.2020.0612 (PMC7063499; doi:10.1001/jamanetworkopen.2020.0612)
Supplement: Supplement. — eMethods. SAS Codes eFigure. Kaplan-Meier Curves for Days From Index Emergency Department Visit to Appendicitis Diagnosis Stratified by Adults and Children With 95% Hall-Wellner Bands eTable 1. Diagnosis and Procedure Codes eTable 2. Percent of Potentially Missed Appendicitis for Demographic and Visit Characteristics eTable 3. Frequency of Clinical Symptoms and Combinations of Symptoms in Patients With Same Day Diagnosis Compared With Those with Potentially Missed Appendicitis eTable 4. Adults—Multivariable Modeling of Potentially Missed Appendicitis With Abdominal Pain and Its Combination With Other Symptom(s) Included Along with Other Covariates (N = 100 833) eTable 5. Children—Multivariable Modeling of Potentially Missed Appendicitis With Abdominal Pain and Its Combination with Other Symptom(s) Included Along With Other Covariates (N = 22 250) eTable 6. Potentially Missed Appendicitis Cases That Had Imaging at Index Emergency Department Visit Compared With Those That Had Imaging on Return Visit [file jamanetwopen-3-e200612-s001.pdf]

Supplementary Online Content

Mahajan P, Basu T, Pai C-W, et al. Factors associated with potentially missed diagnosis of appendicitis in the emergency department. *JAMA Netw Open*. 2020;3(3):e200612. doi:10.1001/jamanetworkopen.2020.0612

eMethods. SAS Codes

eFigure. Kaplan-Meier Curves for Days From Index Emergency Department Visit to Appendicitis Diagnosis Stratified by Adults and Children With 95% Hall-Wellner Bands

eTable 1. Diagnosis and Procedure Codes

eTable 2. Percent of Potentially Missed Appendicitis for Demographic and Visit Characteristics

eTable 3. Frequency of Clinical Symptoms and Combinations of Symptoms in Patients With Same Day Diagnosis Compared With Those with Potentially Missed Appendicitis

eTable 4. Adults—Multivariable Modeling of Potentially Missed Appendicitis With Abdominal Pain and Its Combination With Other Symptom(s) Included Along with Other Covariates (N = 100 833)

eTable 5. Children—Multivariable Modeling of Potentially Missed Appendicitis With Abdominal Pain and Its Combination with Other Symptom(s) Included Along With Other Covariates (N = 22 250)

eTable 6. Potentially Missed Appendicitis Cases That Had Imaging at Index Emergency Department Visit Compared With Those That Had Imaging on Return Visit

This supplementary material has been provided by the authors to give readers additional information about their work.

© 2020 Mahajan P et al. *JAMA Network Open*.

eMethods. SAS Codes

Demographics

```
%Include 'H:\optum\Table\Table.sas';
%Table(dsn=dat.adult_apr,
var =age bus Gdr_Cd RACE_n region comorbidity CT US XRAY cbc_test urine_test,
type=c d d d d d d d d d d,
labelwrap=y,
by=sdd,
outdoc=H:\ED-Prasant\April_2019\Demo_Adult_2019.rtf,
meandec=1,
ttitle1=Demographical Characteristics);
```

Crude odds ratios per symptom

```
%macro od(sym=);
ods pdf body="H:\ED-Prasant\April_2019\Adult\logistic_&sym..pdf";
proc logistic data=dat.adult_apr ;
class &sym(ref="No") /param=ref;
model sdd(event="0")= &sym ;

LABEL NO_ABD="NO ABD";
LABEL S1_ONLY="ABDOMINAL PAIN ONLY";
LABEL ABD_CONS="ABD & CONSTIPATION";
LABEL ABD_VOM="ABD & VOMITING";
LABEL ABD_VOM_FEV="ABD & VOMITING & FEVER";
LABEL ABD_VOM_FEV_CONS="ABD & CONSTIPATION & FEVER & VOMITING";
run;
ods pdf close;
%mend od;

%od(sym=s1_only);
%od(sym=ABD_cons);
%od(sym=ABD_VOM);
%od(sym=ABD_VOM_FEV);
%od(sym=ABD_VOM_FEV_CONS);
%od(sym=NO_abd);
```

Adjusted odds ratios per symptom (along with other covariates)

```
%macro od(sym=);
ods pdf body="H:\ED-Prasant\April_2019\adult\nAdjORs_&sym..pdf";

proc logistic data=dat.adult_apr ;
where GDR_CD ne "U" & region ne "U" ;
class bus region (ref="West") race_n(ref="W")  GDR_CD(ref="M") comorbidity(ref="None")
AGE_f(ref="18 to 25 years") urine_test(ref="No") cbc_test(ref="No") ct(ref="No") us(ref="No") xray(ref="No")
/param=ref;
model sdd(event="0")= &sym AGE_f GDR_CD race_n  region comorbidity  bus  urine_test cbc_test ct us xray
ct*us ct*xray us*xray;
run;
%mend od;
```

© 2020 Mahajan P et al. *JAMA Network Open*.

```
%od(sym=S1_ONLY);
%od(sym=ABD_CONS);
%od(sym=ABD_VOM);
%od(sym=ABD_VOM_FEV);
%od(sym=ABD_VOM_FEV_CONS);
%od(sym=NO_ABD);
```

Adjusted odds ratios stratified by symptom

```
%macro od(sym=);
ods pdf body="H:\ED-Prasant\April_2019\adult\Stratified_&sym..pdf";
proc logistic data=dat.adult_apr ;
where GDR_CD ne "U" & region ne "U" & &sym=1;
class bus region (ref="West") race_n(ref="W") GDR_CD(ref="M") comorbidity(ref="None")
AGE_f(ref="18 to 25 years")   ct(ref="No") us(ref="No") xray(ref="No") urine_test(ref="No")
cbc_test(ref="No")/param=ref;
model sdd(event="0") =  AGE_f GDR_CD race_n  region comorbidity  bus  urine_test cbc_test ct us xray ct*us
ct*xray us*xray;
run;
ods pdf close;
%mend od;

%od(sym=ABD);
%od(sym=VOMITTING);
%od(sym=FEVER);
%od(sym=DIARRHEA);
%od(sym=CONSTIPATION);
```

Survival curves

```
ods pdf file="H:\ED-Prasant\April_2019\Figure2.pdf";
proc lifetest data=dat.data1 atrisk plots=survival(atrisk cb) timelist=0 to 30 by 2;
strata age_cat;
time day*sdd(1);
label day="Days Since Index ED Visit";
label age_cat="Study population";
format age_cat adult.  ;
run;
ods pdf close;
```

© 2020 Mahajan P et al. *JAMA Network Open*.

eFigure. Kaplan-Meier Curves for Days From Index Emergency Department Visit to Appendicitis Diagnosis Stratified by Adults and Children With 95% Hall-Wellner Bands

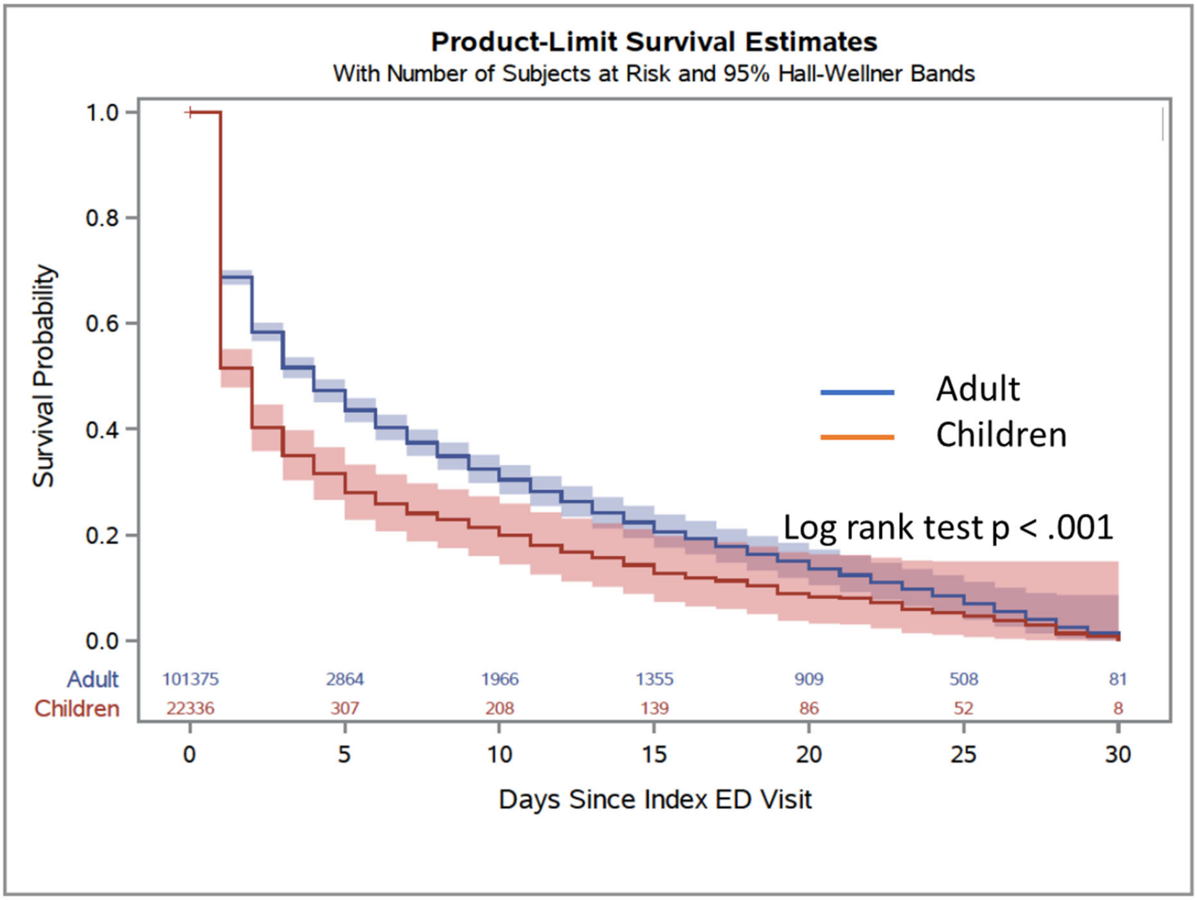

ED indicates emergency department.

The y-axis represents the appendicitis-free survival probability in potentially missed appendicitis cohort stratified by children and adults, while the x-axis represents the number of days following the index ED visit. The log rank test is used as a basis to determine if the survival curves are different (by the strata).

© 2020 Mahajan P et al. *JAMA Network Open*.

eTable 1. Diagnosis and Procedure Codes

	ICD-9-CM	*ICD-10-CM*	*CPT-4*	DRG
**Diagnostic Code**				
Appendicitis	540<br>541<br>542<br>543	K35<br>K36<br>K37<br>K38		
Abdominal pain	789.0	R10		
Vomiting/nausea	787.0	R11		
Diarrhea	787.91	R19.7		
Constipation	564.0	K59.0		
Fever	780.60	R50.9		
**Procedure Code**				
Appendectomy	47.0<br>47.1<br>47.2<br>47.9<br>54.1	0D5J0ZZ<br>0D5J3ZZ<br>0D5J4ZZ<br>0D5J7ZZ<br>0D5J8ZZ<br>0D9J00Z<br>0D9J0ZX<br>0D9J0ZZ<br>0D9J30Z<br>0D9J3ZX<br>0D9J3ZZ<br>0D9J40Z<br>0D9J4ZX<br>0D9J4ZZ<br>0D9J70Z<br>0D9J7ZX<br>0D9J7ZZ<br>0D9J80Z<br>0D9J8ZX<br>0D9J8ZZ<br>0DBJ0ZX<br>0DBJ0ZZ<br>0DBJ3ZX<br>0DBJ3ZZ<br>0DBJ4ZX<br>0DBJ4ZZ<br>0DBJ7ZX<br>0DBJ7ZZ<br>0DBJ8ZX<br>0DBJ8ZZ<br>0DCJ0ZZ<br>0DCJ3ZZ<br>0DCJ4ZZ<br>0DCJ7ZZ<br>0DCJ8ZZ<br>0DFJ0ZZ<br>0DFJ3ZZ<br>0DFJ4ZZ	44900<br>44901<br>44950<br>44955<br>44960<br>44970<br>44979	338<br>339<br>340<br>341<br>342<br>343

© 2020 Mahajan P et al. *JAMA Network Open*.

		0DFJ7ZZ<br>0DFJ8ZZ<br>0DNJ7ZZ<br>0DNJ8ZZ		
		0DQJ0ZZ<br>0DQJ3ZZ<br>0DQJ4ZZ<br>0DQJ7ZZ<br>0DQJ8ZZ<br>0DTJ0ZZ<br>0DTJ4ZZ<br>0DTJ7ZZ<br>0DTJ8ZZ		
Urinalysis			81001<br>81002<br>81003<br>81005	
CBC			85025<br>85027	
Abdominal CT			74150<br>74160<br>74170<br>74174<br>74175<br>74176<br>74177<br>74178	
Abdominal US			76700<br>76705	
Abdominal x-ray			74000<br>74010<br>74020<br>74022	

Abbreviations: CBC, complete blood count; *CPT-4*, *Current Procedural Terminology, 4th Edition*; CT, computed tomography; DRG, Diagnosis-Related Group; *ICD-9-CM*, *International Classification of Diseases, 9th Revision, Clinical Modification*; *ICD-10-CM*, *International Statistical Classification of Diseases and Related Health Problems, 10th Revision*; US, Ultrasonography.

© 2020 Mahajan P et al. *JAMA Network Open*.

eTable 2. Percent of Potentially Missed Appendicitis for Demographic and Visit Characteristics

	Adult					Children			
		Potentially Missed Appendicitis					Potentially Missed Appendicitis		
	All	No.	%	*P* Value		All	No.	%	*P* Value
Sex									
Female	51923	3884	7.5%	<.001		9631	533	5.5%	<.001
Male	49452	2176	4.4%			12705	440	3.5%	
Race						0			
White	56672	3473	6.1%	<.001		12875	594	4.6%	.05
Asian	3206	135	4.2%			713	29	4.1%	
Black	6435	506	7.9%			1044	53	5.1%	
Hispanic	11400	643	5.6%			3331	118	3.5%	
Unknown	23652	1303	5.5%			4373	179	4.1%	
Comorbidity index									
0	48282	1468	3.0%	<.001		17108	598	3.5%	<.001
1	20529	1078	5.3%			4029	254	6.3%	
2 or higher	32564	3514	10.8%			1199	121	10.1%	
Imaging study									
CT	93424	5183	5.5%	<.001		13399	630	4.7%	.002
US	10032	1354	13.5%	<.001		9764	468	4.8%	.005
X-ray	11880	1693	14.3%	<.001		4384	367	8.4%	<.001
Lab test									
Urinalysis	21396	1592	7.4%	<.001		3716	217	5.8%	<.001
CBC	22856	2032	8.9%	<.001		3281	222	6.8%	<.001
Census region									
Midwest	24804	1521	6.1%	.06		5384	269	5.0%	.026
Northeast	9292	582	6.3%			1976	70	3.5%	
South	44876	2583	5.8%			9887	435	4.4%	
West	21878	1335	6.1%			5004	195	3.9%	
Unknown	525	39	7.4%			85	4	4.7%	
Insurance									
Commercial	82552	4066	4.9%	<.001		22336	973	4.4%	--
Medicare	18823	1994	10.6%			--	--	--	--

Abbreviations: CBC, complete blood count ; CT, computed tomography; US, ultrasonography.

For race and ethnicity, we used the definitions from the Clinformatics DataMart, where there is only one race category and each appear mutually exclusive. They use a proprietary algorithm representing a compilation of fields including known race or proprietary ethnic code tables. This is a combination of information sources including public records, self-reported surveys, and a proprietary ethnic code table.

© 2020 Mahajan P et al. *JAMA Network Open*.

eTable 3. Frequency of Clinical Symptoms and Combinations of Symptoms in Patients With Same Day Diagnosis Compared With Those with Potentially Missed Appendicitis

	**Adult, No. (%)**				**Children, No. (%)**		
	**Same Day Diagnosis**	**Potentially Missed Appendicitis**			**Same Day Diagnosis**	**Potentially Missed Appendicitis**	
	**(N=95,315)**	**(N=6,060)**	***P* Value**		**(N=21,363)**	**(N=973)**	***P* Value**
Any individual symptom							
Abdominal pain	93,285 (97.9)	5487 (90.5)	<.001		20,948 (98.1)	906 (93.1)	<.001
Vomiting/nausea	29,768 (31.2)	2126 (35.1)	<.001		8,598 (40.2)	420 (43.2)	.07
Fever	6,272 (6.6)	552 (9.1)	<.001		3,619 (16.9)	182 (18.7)	.15
Diarrhea	6,292 (6.6)	740 (12.2)	<.001		1,597 (7.5)	103 (10.6)	<.001
Constipation	4,014 (4.2)	548 (9.0)	<.001		1,541 (7.2)	140 (14.4)	<.001
Abdominal pain only	56,644 (59.4)	2,811 (46.4)	<.001		10,003 (46.8)	374 (38.4)	<.001
No abdominal pain at all	2,030 (2.1)	573 (9.5)	<.001		415 (1.9)	67 (6.9)	<.001
Combination of symptoms							
Abdominal pain + vomiting/nausea	21,154 (22.2)	1,195 (19.7)	<.001		5,140 (24.1)	199 (20.5)	.01
Abdominal pain + fever	2,748 (2.9)	177 (2.9)	.86		1,268 (5.9)	51 (5.2)	.37
Abdominal pain + diarrhea	1,715 (1.8)	184 (3.0)	<.001		264 (1.2)	15 (1.5)	.40
Abdominal pain + constipation	1,953 (2.0)	228 (3.8)	<.001		636 (3.0)	67 (6.9)	<.001
Abdominal pain + vomiting/nausea + fever	2,103 (2.2)	103 (1.7)	.009		1,434 (6.7)	55 (5.7)	.19

© 2020 Mahajan P et al. *JAMA Network Open*.

eTable 4. Adults—Multivariable Modeling of Potentially Missed Appendicitis With Abdominal Pain and Its Combination With Other Symptom(s) Included Along with Other Covariates (N = 100 833)

	Adjusted Odds Ratio (95% CI)		
	Abd Pain Only	Abd Pain + Constipation	Abd Pain + Nausea/Vomiting
Symptom	0.65 (0.62-0.69)	1.51 (1.31-1.75)	0.90 (0.84-0.97)
Age group (reference=18-25)			
26 - 40	1.01 (0.92-1.11)	1.00 (0.91-1.09)	0.99 (0.91-1.09)
41 - 64	0.86 (0.79-0.95)	0.84 (0.77-0.92)	0.83 (0.76-0.92)
65 or older	0.91 (0.80-1.04)	0.88 (0.77-1.00)	0.87 (0.76-0.99)
Sex (reference=male)			
Female	1.56 (1.47-1.64)	1.59 (1.51-1.68)	1.60 (1.51-1.69)
Race (reference=white)			
Asian	0.75 (0.62-0.89)	0.74 (0.62-0.88)	0.74 (0.62-0.89)
Black	1.15 (1.04-1.27)	1.15 (1.04-1.28)	1.16 (1.05-1.28)
Hispanic	0.96 (0.88-1.06)	0.96 (0.87-1.05)	0.96 (0.87-1.05)
Unknown/missing	0.93 (0.87-1.00)	0.93 (0.87-1.00)	0.93 (0.87-1.00)
Comorbidity Index (reference=0)			
1	1.72 (1.58-1.86)	1.73 (1.59-1.88)	1.73 (1.60-1.88)
2 or higher	3.43 (3.18-3.69)	3.53 (3.28-3.80)	3.54 (3.29-3.81)
Imaging study (reference=no imaging study)			
Any CT	0.51 (0.46-0.57)	0.51 (0.46-0.56)	0.51 (0.46-0.57)
Any US, no CT	1.94 (1.65-2.29)	1.92 (1.63-2.26)	1.92 (1.63-2.26)
X-ray only	2.52 (1.99-3.18)	2.60 (2.06-3.28)	2.66 (2.11-3.35)
Insurance (reference=Medicare)			
Commercial	0.79 (0.72-0.88)	0.78 (0.71-0.87)	0.78 (0.70-0.86)
Census region (reference=West)			
Midwest	1.10 (1.02-1.19)	1.10 (1.02-1.19)	1.10 (1.02-1.19)
Northeast	0.99 (0.90-1.10)	1.01 (0.91-1.12)	1.01 (0.91-1.12)
South	0.94 (0.88-1.01)	0.96 (0.90-1.03)	0.96 (0.90-1.03)
Lab test (reference=respective lab not performed)			
Urinalysis	0.93 (0.87-1.00)	0.92 (0.86-0.99)	0.92 (0.86-0.99)
CBC	1.37 (1.28-1.47)	1.39 (1.29-1.49)	1.39 (1.29-1.49)
C-statistic	0.710	0.704	0.703
Symptom	0.78 (0.64-0.95)	0.94 (0.47-1.87)	3.57 (3.22-3.95)
Age group (reference=18-25)			
26 - 40	0.99 (0.91-1.09)	0.99 (0.91-1.09)	0.99 (0.90-1.09)
41 - 64	0.84 (0.77-0.92)	0.84 (0.77-0.92)	0.83 (0.76-0.91)
65 or older	0.88 (0.77-1.00)	0.88 (0.77-1.00)	0.84 (0.74-0.96)
Sex (reference=male)			

© 2020 Mahajan P et al. *JAMA Network Open*.

Female	1.59 (1.51-1.68)	1.59 (1.51-1.68)	1.62 (1.53-1.71)
Race (reference=white)			
Asian	0.74 (0.62-0.89)	0.74 (0.62-0.89)	0.74 (0.62-0.89)
Black	1.16 (1.05-1.28)	1.16 (1.05-1.28)	1.14 (1.03-1.26)
Hispanic	0.96 (0.87-1.05)	0.96 (0.87-1.05)	0.95 (0.87-1.04)
Unknown/missing	0.94 (0.87-1.00)	0.94 (0.87-1.00)	0.93 (0.87-1.00)
Comorbidity Index (reference=0)			
1	1.73 (1.60-1.88)	1.73 (1.60-1.88)	1.73 (1.59-1.88)
2 or higher	3.54 (3.29-3.81)	3.54 (3.29-3.81)	3.44 (3.19-3.70)
Imaging study (reference=no any imaging study)			
Any CT	0.51 (0.46-0.57)	0.51 (0.46-0.57)	0.56 (0.50-0.62)
Any US, no CT	1.92 (1.63-2.26)	1.92 (1.63-2.26)	2.08 (1.77-2.46)
X-ray only	2.64 (2.10-3.33)	2.65 (2.10-3.34)	2.69 (2.13-3.41)
Insurance (reference=Medicare)			
Commercial	0.78 (0.70-0.86)	0.78 (0.70-0.86)	0.80 (0.73-0.89)
Census region (reference=West)			
Midwest	1.10 (1.02-1.19)	1.10 (1.02-1.19)	1.09 (1.00-1.18)
Northeast	1.01 (0.91-1.12)	1.01 (0.91-1.12)	1.00 (0.90-1.11)
South	0.96 (0.90-1.03)	0.96 (0.90-1.03)	0.96 (0.89-1.03)
Lab test (reference=respective lab not performed)			
Urinalysis	0.92 (0.86-0.99)	0.92 (0.86-0.99)	0.95 (0.88-1.02)
CBC	1.39 (1.30-1.49)	1.39 (1.29-1.49)	1.37 (1.28-1.47)
C-statistic	0.703	0.703	0.711

Abbreviations: Abd, abdominal; CBC, complete blood count; CT, computed tomography; US, ultrasonography.

For adjusted odds ratios with presence of symptom, referent groups include all others that were not in this undifferentiated symptom category. For example, patient episodes that presented with "'abdominal pain only'" would have a referent group of all others that were not "'abdominal pain only'.

For race and ethnicity, we used the definitions from the Clinformatics DataMart, where there is only one race category and each appear mutually exclusive. They use a proprietary algorithm representing a compilation of fields including known race or proprietary ethnic code tables. This is a combination of information sources including public records, self-reported surveys, and a proprietary ethnic code table.

© 2020 Mahajan P et al. *JAMA Network Open*.

eTable 5. Children—Multivariable Modeling of Potentially Missed Appendicitis With Abdominal Pain and Its Combination with Other Symptom(s) Included Along With Other Covariates (N = 22 250)

	Adjusted Odds Ratio (95% CI)		
	Abd Pain Only	Abd Pain + Constipation	Abd Pain + Nausea/Vomiting
Symptom	0.79 (0.69-0.90)	2.43 (1.86-3.17)	0.84 (0.71-0.98)
Age group (reference=0-5)			
6 – 10	0.50 (0.39-0.63)	0.53 (0.41-0.68)	0.49 (0.38-0.62)
11 - 15	0.52 (0.41-0.65)	0.47 (0.37-0.60)	0.50 (0.40-0.63)
16 - 17	0.56 (0.44-0.72)	0.49 (0.39-0.61)	0.54 (0.42-0.69)
Sex (reference=male)			
Female	1.60 (1.41-1.83)	1.61 (1.41-1.83)	1.61 (1.41-1.84)
Race (reference=white)			
Asian	0.92 (0.62-1.35)	0.91 (0.62-1.34)	0.92 (0.62-1.35)
Black	1.08 (0.80-1.45)	1.07 (0.79-1.43)	1.08 (0.81-1.45)
Hispanic	0.76 (0.62-0.94)	0.76 (0.62-0.94)	0.76 (0.61-0.93)
Unknown/missing	0.91 (0.76-1.08)	0.90 (0.76-1.08)	0.91 (0.76-1.08)
Comorbidity Index (reference=0)			
1	1.78 (1.53-2.07)	1.80 (1.55-2.10)	1.81 (1.55-2.10)
2 or higher	2.74 (2.22-3.38)	2.85 (2.31-3.52)	2.85 (2.31-3.51)
Imaging study (reference=no any imaging study)			
Any CT	1.00 (0.80-1.24)	0.98 (0.79-1.23)	1.01 (0.81-1.25)
Any US, no CT	0.74 (0.58-0.95)	0.71 (0.56-0.91)	0.74 (0.58-0.94)
X-ray only	1.74 (1.16-2.59)	1.54 (1.03-2.32)	1.75 (1.17-2.62)
Census region (reference=West)			
Midwest	1.21 (0.99-1.46)	1.19 (0.98-1.45)	1.19 (0.98-1.45)
Northeast	0.92 (0.69-1.22)	0.90 (0.68-1.20)	0.91 (0.69-1.21)
South	1.09 (0.91-1.29)	1.09 (0.91-1.30)	1.11 (0.93-1.32)
Lab test (reference=respective lab not performed)			
Urinalysis	1.06 (0.88-1.27)	1.05 (0.88-1.27)	1.06 (0.88-1.27)
CBC	1.58 (1.32-1.90)	1.60 (1.33-1.92)	1.58 (1.32-1.90)
C-statistic	0.657	0.665	0.655
Symptom	0.77 (0.58-1.02)	1.09 (0.56-2.10)	2.99 (2.25-3.96)
Age group (reference=0–5)			
6 – 10	0.48 (0.38-0.61)	0.48 (0.38-0.62)	0.54 (0.42-0.69)
11 – 15	0.48 (0.38-0.60)	0.49 (0.39-0.61)	0.55 (0.44-0.70)
16 – 17	0.51 (0.40-0.66)	0.53 (0.41-0.68)	0.60 (0.46-0.77)
Sex (reference=male)			
Female	1.61 (1.41-1.84)	1.61 (1.41-1.84)	1.62 (1.42-1.85)
Race (reference=white)			
Asian	0.91 (0.62-1.35)	0.92 (0.62-1.35)	0.93 (0.63-1.36)

© 2020 Mahajan P et al. *JAMA Network Open*.

Black	1.08 (0.81-1.45)	1.08 (0.81-1.45)	1.07 (0.80-1.44)
Hispanic	0.76 (0.61-0.93)	0.76 (0.62-0.93)	0.76 (0.61-0.93)
Unknown/missing	0.91 (0.76-1.08)	0.91 (0.76-1.08)	0.91 (0.76-1.08)
Comorbidity Index (reference=0)			
1	1.81 (1.55-2.11)	1.80 (1.55-2.10)	1.78 (1.53-2.08)
2 or higher	2.86 (2.32-3.53)	2.84 (2.31-3.50)	2.64 (2.14-3.27)
Imaging study (reference=no any imaging study)			
Any CT	1.01 (0.81-1.26)	1.01 (0.81-1.25)	1.06 (0.85-1.32)
Any US, no CT	0.74 (0.58-0.95)	0.74 (0.58-0.95)	0.77 (0.60-0.99)
X-ray only	1.76 (1.18-2.63)	1.77 (1.19-2.64)	1.78 (1.19-2.66)
Census region (reference=West)			
Midwest	1.19 (0.98-1.45)	1.20 (0.98-1.45)	1.17 (0.96-1.42)
Northeast	0.91 (0.69-1.21)	0.92 (0.69-1.21)	0.91 (0.69-1.21)
South	1.11 (0.93-1.32)	1.10 (0.92-1.31)	1.09 (0.91-1.30)
Lab test (reference=respective lab not performed)			
Urinalysis	1.06 (0.88-1.27)	1.06 (0.88-1.27)	1.08 (0.90-1.30)
CBC	1.59 (1.32-1.91)	1.59 (1.32-1.91)	1.57 (1.31-1.89)
C-statistic	0.654	0.654	0.656

Abbreviations: Abd, abdominal; CBC, complete blood count; CT, computed tomography; US, ultrasonography.

For adjusted odds ratios with presence of symptom, referent groups include all others that were not in this undifferentiated symptom category. For example, patient episodes that presented with "'abdominal pain only'" would have a referent group of all others that were not '"abdominal pain only'.

For race and ethnicity, we used the definitions from the Clinformatics DataMart, where there is only one race category and each appear mutually exclusive. They use a proprietary algorithm representing a compilation of fields including known race or proprietary ethnic code tables. This is a combination of information sources including public records, self-reported surveys, and a proprietary ethnic code table.

© 2020 Mahajan P et al. *JAMA Network Open*.

eTable 6. Potentially Missed Appendicitis Cases That Had Imaging at Index Emergency Department Visit Compared With Those That Had imaging on Return Visit

	Children			Adult		
	Index ED Visit No. (%)	Return Visit No. (%)	No. Total	Index ED Visit No. (%)	Return Visit No. (%)	No. Total
CT	13 (2.06)	617 (97.94)	630	105 (2.03)	5078 (97.97)	5183
US	13 (2.78)	455 (97.22)	468	78 (5.76)	1276 (94.24)	1354
X-ray	23 (6.27)	344 (93.73)	367	167 (9.86)	1526 (90.14)	1693
CBC	17 (7.66)	205 (92.34)	222	138 (6.79)	1894 (93.21)	2032
Urine	5 (2.30)	212 (97.7)	217	5 (4.59)	1519 (95.41)	1592

Abbreviations: CBC, complete blood count; CT, computed tomography; ED, emergency department; US, ultrasonography.

© 2020 Mahajan P et al. *JAMA Network Open*.
